# Possible Incidental Parkinson’s Disease following Asthma: A Nested Case–Control Study in Korea

**DOI:** 10.3390/jpm13050718

**Published:** 2023-04-24

**Authors:** Mi Jung Kwon, Joo-Hee Kim, Ho Suk Kang, Hyun Lim, Min-Jeong Kim, Nan Young Kim, Se Hoon Kim, Hyo Geun Choi, Eun Soo Kim

**Affiliations:** 1 Department of Pathology, Division of Neuropathology, Hallym University Sacred Heart Hospital, Hallym University College of Medicine, Anyang 14068, Republic of Korea; mulank@hanmail.net; 2 Laboratory of Brain and Cognitive Sciences for Convergence Medicine, Hallym University College of Medicine, Anyang 14068, Republic of Korea; 3 Division of Pulmonary, Allergy, and Critical Care Medicine, Department of Medicine, Hallym University Sacred Heart Hospital, Hallym University College of Medicine, Anyang 14068, Republic of Korea; luxjhee@hallym.or.kr; 4 Division of Gastroenterology, Department of Internal Medicine, Hallym University Sacred Heart Hospital, Hallym University College of Medicine, Anyang 14068, Republic of Korea; hskang76@hallym.or.kr (H.S.K.); hlim77@hallym.or.kr (H.L.); 5 Department of Radiology, Hallym University Sacred Heart Hospital, Hallym University College of Medicine, Anyang 14068, Republic of Korea; drkmj@naver.com; 6 Hallym Institute of Translational Genomics and Bioinformatics, Hallym University Medical Center, Anyang 14068, Republic of Korea; honeyny78@gmail.com; 7 Department of Pathology, Division of Neuropathology, Severance Hospital, Yonsei University College of Medicine, Seoul 03722, Republic of Korea; paxco@yuhs.ac; 8 Suseo Seoul E.N.T. Clinic and MD Analytics, 10, Bamgogae-ro 1-gil, Gangnam-gu, Seoul 06349, Republic of Korea

**Keywords:** Parkinson’s disease, asthma, nested case–control study, epidemiologic study

## Abstract

A connection between asthma and the occurrence of Parkinson’s disease (PD) has been suggested, but the findings have been contentious and require verification. In this nested case–control study using data from the Korean National Health Insurance Service—Health Screening Cohort (2002–2019), which comprised 9029 participants with PD and 36,116 matched controls, we explored the relationship between asthma and incident PD. An overlap-weighted logistic regression model was used to measure the probability of asthma and PD. After adjusting for various covariates, we found that asthma was related to a 1.11-fold greater probability of PD (95% confidence interval: 1.06–1.16). A subgroup analysis showed that this effect was independent of age, sex, residential area, or alcohol consumption, and that it was still noticeable even among patients with a high income; those with a normal weight or obesity; those who were non-smokers or current smokers; and those with no history of chronic obstructive pulmonary disease, hypertension, hyperglycemia, hyperlipidemia, or anemia. Thus, these findings may indicate that asthma may slightly augment the likelihood of PD in the Korean adult population regardless of demographic or lifestyle factors, making it difficult to predict PD in asthma patients.

## 1. Introduction

Parkinson’s disease (PD) and asthma are more prevalent diseases in older adults, with increasing incidence rates as people age. PD, the second most common neurodegenerative disorder, has the highest incidence rates occurring in people over the age of 60 [1] and presents with four cardinal motor signs: rigidity, bradykinesia, tremor at rest, and postural instability [2]. In the Republic of Korea, the age- and gender-standardized prevalence has grown from 115.9 to 139.8 cases per 100,000 people from 2010 to 2015, with a yearly incidence of 23.9 cases per 100,000 people [3]. This increase is greater than what has been seen in other countries; the yearly incidence increased by 1.17 times in the USA between 1986–1995 and 1996–2005 [4], 0.97 times in Taiwan between 2005 and 2011 [5], 0.60 times in the UK between 1999 and 2009 [6], and 0.36 times in the Netherlands between the 1990s and 2000s [7]. On the other hand, asthma has a prevalence of 2–20% [8], which varies by ethnic group [9]. The incidence of asthma tends to primarily peak in childhood and early adulthood, and there is a second peak in incidence that occurs in older adults, particularly in those aged 65 years and above [10]. The incidence of asthma is increasing, particularly in Western-lifestyle-dominant areas [11]. In the Republic of Korea, the incidence of asthma is estimated to be 6.07 per 1000 people [12]. With the aging population and changing lifestyles in the Republic of Korea, PD and asthma have become a major public health concern [3], making it a financial and health burden to society [13,14]. 

Recent studies have suggested that asthma may increase the risk of developing PD, with Taiwanese population-based studies finding a 3.10-fold higher risk of PD in asthmatic patients aged > 45 years [15] (95% confidence interval [CI]: 2.20–4.36), and a 1.56-fold higher risk of PD in asthmatic patients who have concomitant chronic obstructive pulmonary disease (COPD) and are aged > 40 years (95% CI: 1.34–1.81) [16]. PD causes α-synucleinopathy in dopaminergic neurons of the substantia nigra, which progressively leads to central nervous system dysfunction [2,17]. In contrast, while type 2 helper T cell-related asthma is one possible cause [18], other physiological systems have been identified as contributing to the development of asthma in association with chronic diseases such as hypertension [19], diabetes [20], dyslipidemia [20,21], coronary heart disease [21], and depression [15,22]. Therefore, it may be of great clinical significance to explore whether PD could develop in association with asthma, as another related medical comorbidity. Experimental evidence has supported their potential association based on the shared mechanisms of inflammation, oxidative stress, and immune dysfunction that contribute to the development of both conditions [15,23]. For example, the activation of NF-kB by viral or environmental toxins, oxidative stress, inflammation, and cytokines could be a critical link between asthma and PD [24,25]. NF-kB regulates the expression of genes involved in both conditions, including enzymes, cytokines (such as interleukin(IL)-1, IL-6, IL-8, chemokines, and tumor necrosis factor (TNF)) [26], which are involved in both asthma and PD [24,25]. Type 2 helper T cell-related chronic inflammation associated with asthma may spread to the brain, causing neuroinflammatory cascades and the presence of activated microglia and astroglia [27]. This leads to the production of proinflammatory cytokines (e.g., TNF-α, IL-1β, IL-6) and enzymes (e.g., nicotinamide adenine dinucleotide phosphate oxidase and cyclooxygenase-2) that affect the central nervous system microenvironment, speeding up PD pathogenesis [28].

Previous studies were based on young and middle-aged adult populations [15,16]; it is still unclear if asthma raises the risk of PD in older people, who have a higher prevalence of PD. Additionally, the control groups in previous studies were matched to asthma patients based on age and sex only, and the asthma group had a higher rate of comorbidities, such as diabetes, hypertension, and hyperlipidemia [15,16], thus requiring further validation using large population cohorts with balanced demographics. 

We conducted a nested case–control study utilizing Republic of Korea national public healthcare data to examine the potential relationship between asthma and the emergence of incident PD. We speculated that factors such as age, sex, income, residential area, weight, smoking status, fasting blood glucose level, alcohol consumption, blood pressure, total cholesterol level, hemoglobin level, comorbidities, and COPD history may affect the emergence of incident PD. In an era of personalized medicine, this discovery may help in understanding the emergence of incident PD and its possible prevention measures for individuals who have asthma.

## 2. Materials and Methods

### 2.1. Study Population

The ethics committee of Hallym University (IRB No: 2019-10-023) granted approval for this study in accordance with the IRB’s regulations. For our research, we utilized the data from the Korean National Health Insurance Service—Health Screening Cohort, which offers population-based electronic files for research purposes that were deidentified in the identification codes with anonymous information of the Korean population [29,30,31]. The diagnostic codes of the Korean National Health Insurance Service—Health Screening Cohort data follow the International Classification of Diseases, 10th Revision, Clinical Modification (ICD-10-CM).

### 2.2. Parkinson’s Disease (Outcome)

To ensure an accurate diagnosis, we only included participants who had visited the clinic on multiple occasions and had been classified using ICD-10 codes (G20) for Parkinson’s disease.

### 2.3. Asthma (Exposure)

We looked for individuals who had been officially diagnosed with either asthma (ICD-10: J45) or status asthmaticus (J46) between 2002 and 2019. These people had to have been identified as having asthma at least twice by a doctor and have been treated with medication for asthma, such as inhaled corticosteroids with long-acting β2-agonists, oral leukotriene antagonists, short-acting β2-agonists, systemic LABAs, xanthine derivatives, or systemic corticosteroids.

### 2.4. Participant Selection

**Figure 1:**
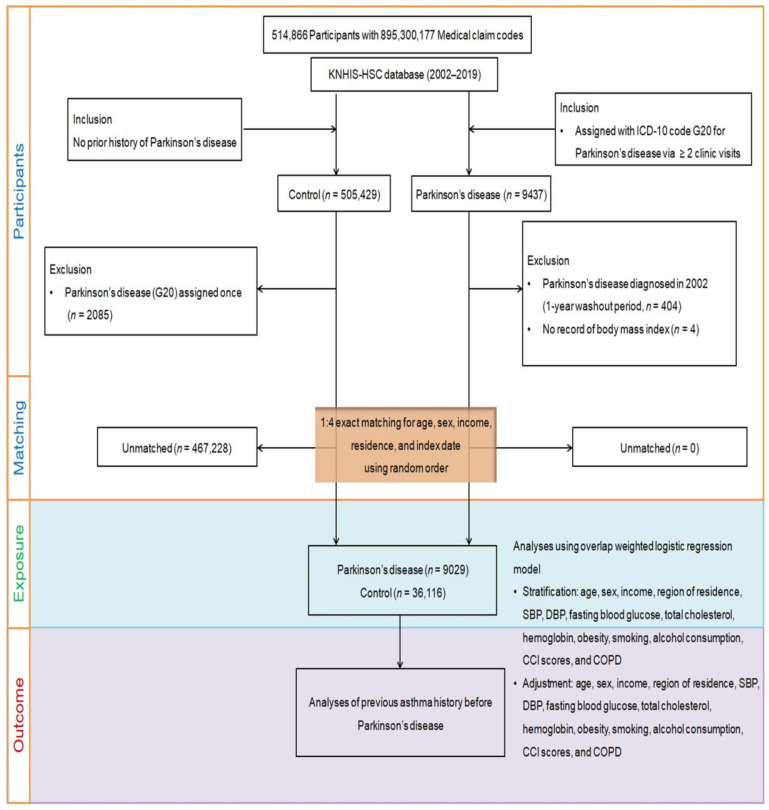
A diagram depicting the process of participant selection that was employed for this research. Out of a total of 514,866 individuals, 9029 were diagnosed with Parkinson’s disease, and 36,116 served as controls, with the two groups being matched at a ratio of 1:4 based on age, gender, income, and place of residence.

This is a retrospective, nested case–control study, using data from the Korean National Health Insurance Service—Health Screening Cohort. Participants with PD (n = 9437) and 505,429 without PD in the control group were selected from 514,866 adult patients (age ≥ 40 years) with 895,300,177 medical claim codes based on the Korean National Health Insurance Service—Health Screening Cohort database from 2002 to 2019 (Figure 1). We excluded the patients diagnosed with PD in 2002 (1-year washout period, n = 404), as we possibly might include pre-existing PD before the index date in the analysis. In addition, four patients lacking body mass index (BMI) records were also excluded. Additionally, 2085 control participants who had been diagnosed with PD were removed. To minimize selection bias, a 1:4 match was made between the remaining PD participants (n = 9029) and 36,116 control participants in terms of age, sex, income, and region of residence in a random number order. The index date for PD participants was the time of their treatment, and that for control participants was the time of their PD match. Consequently, 467,228 control participants were not included in the matching process, leaving 9029 PD participants and 36,116 control participants to be matched.

### 2.5. Covariates

The age groups were split into 10 intervals of five years. The income groups were classified into five categories from the lowest to the highest. The region of residence was divided into rural and urban sectors based on a prior study [32]. Smoking, alcohol consumption, and obesity (measured by BMI) were classified according to a previous study [33]. Systolic (SBP, mmHg) and diastolic (DBP, mmHg) blood pressure, fasting blood glucose (mg/dL), total cholesterol (mg/dL), and hemoglobin (g/dL) levels were recorded. The Charlson Comorbidity Index (CCI) was used to gauge the burden of disease based on 17 comorbidities, with 0 signifying no comorbidities and 29 representing multiple comorbidities [34,35]. Respiratory illnesses were excluded from the CCI score. COPD was assigned for asthma and defined as emphysema (J43), other COPD (J44) except MacLeod syndrome (J430) ≥ 2 times, and the use of long-acting muscarinic antagonists, long-acting β2-agonists, inhaled corticosteroids in combination with long-acting β2-agonists, short-acting muscarinic antagonists, short-acting β2-agonists, methylxanthines, prostaglandin E4 inhibitors, and systemic β-agonists [36].

### 2.6. Statistical Analyses

Overlap weighting with propensity scores was used to adjust for covariate balance and sample size. A propensity score was calculated using a multivariable logistic regression model with all covariates. PD participants were weighted according to the probability of PS, and control participants were weighted according to 1—propensity score (ranging from 0 to 1). This method enabled exact balance and improved precision [37,38,39]. The differences in general characteristics between PD and control groups were then compared with standardized differences before and after weighting. 

Propensity score overlap-weighted multivariable logistic regressions for crude (unadjusted) and overlap-weighted (adjusted for all covariates) models were used to estimate overlap-weighted ORs and 95% CIs of asthma for incident PD by adjusting for potential confounders. Subgroup analyses were performed according to all covariates. Two-tailed analyses were used, and significance was set at *p* < 0.05. Statistical analyses were conducted using SAS version 9.4 (SAS Institute Inc., Cary, NC, USA).

## 3. Results

### 3.1. Baseline Characteristics

This study included 9029 participants with PD and 36,116 propensity score-matched controls. Table 1 displays the baseline characteristics of both cohorts prior to and after overlap-weighted adjustment. The PD and comparison groups were precisely matched, making their demographics (sex, age, region, and income) the same (standardized difference = 0). Moreover, extra characteristics, including weight, smoking status, alcohol consumption, SBP, fasting blood glucose, DBP, total cholesterol, hemoglobin, CCI score, and COPD history, were comparable among the PD and control groups (standardized difference ≤ 0.2) apart from the CCI score (standardized difference = 0.27) before overlap-weighted adjustment. After adjusting for discrepancies through overlap weighting, the standardized mean differences between the two cohorts decreased to a near-perfect balance.

### 3.2. Association of Asthma with Parkinson’s Disease 

**Figure 2:**
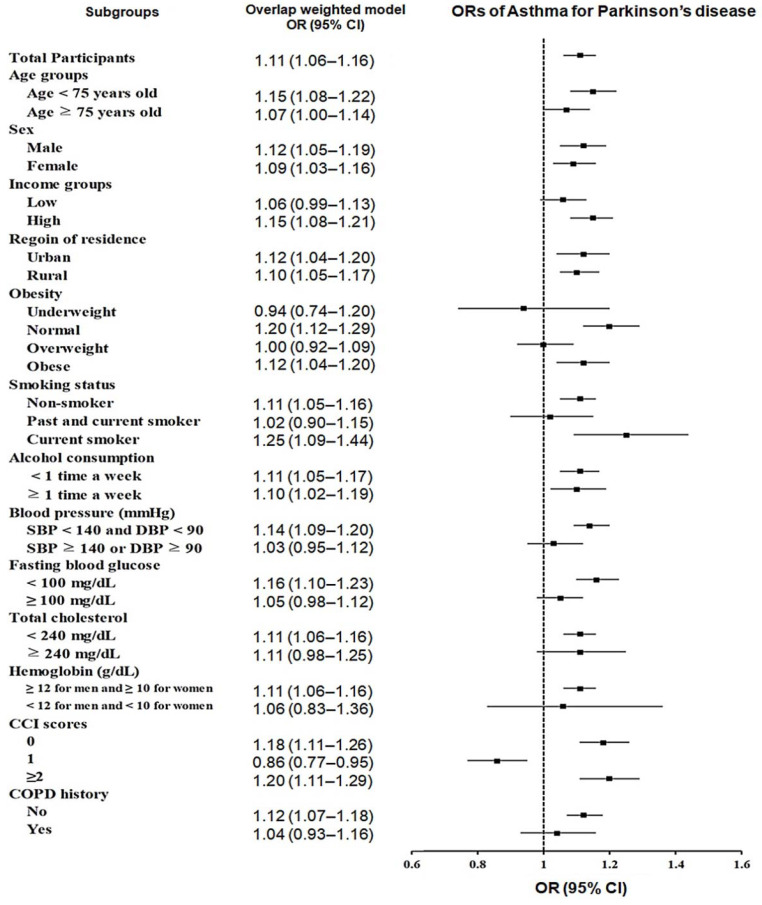
Forest plots depicting the association between asthma and subsequent probability of incident Parkinson’s disease according to subgroups.

We investigated the potential connection between asthma and PD compared to the control group (Table 2). The results showed that asthma was correlated with a small, increased risk of PD (adjusted odds ratio [aOR], 1.11; 95% CI, 1.06–1.16; *p <* 0.001). To further investigate this association, subgroup examinations were performed (Figure 2). Patients were categorized by age, gender, income, and residential area to identify the association between asthma and PD. No matter the age (<75 years: aOR, 1.15; 95% CI, 1.08–1.22; *p <* 0.001 vs. ≥75 years: aOR, 1.07; 95% CI, 1.00–1.14; *p =* 0.041), sex (male: aOR, 1.12; 95% CI, 1.05–1.19; *p =* 0.001 vs. female: aOR, 1.09; 95% CI, 1.03–1.16; *p =* 0.002), or location (urban: aOR, 1.12; 95% CI, 1.04–1.20; *p* = 0.003 vs. rural: aOR, 1.10; 95% CI, 1.05–1.17; *p <* 0.001), the correlation between asthma and PD remained conspicuous. In terms of economic income, the incidence of PD was significantly higher in patients with asthma and a high income (aOR, 1.15; 95% CI, 1.08–1.21; *p <* 0.001), although this association was not observed in those with a low income (aOR, 1.06; 95% CI, 0.99–1.13; *p =* 0.101).

In further subgroup analyses (Table 3), having asthma was connected to an unremarkable yet steady increase in the probability of PD in people with normal weight (aOR, 1.20; 95% CI, 1.12–1.29; *p <* 0.001) or obesity (BMI 25–30; aOR, 1.12; 95% CI, 1.04–1.20; *p =* 0.003); those who were non-smokers (aOR, 1.11; 95% CI, 1.05–1.16; *p <* 0.001) or smokers (aOR, 1.25; 95% CI, 1.09–1.44; *p =* 0.002); those who drank alcohol more than once a week (aOR, 1.11; 95% CI, 1.05–1.17; *p <* 0.001) or less than once a week (aOR, 1.10; 95% CI, 1.02–1.19; *p =* 0.020); those who had a SBP < 140 mmHg and a DBP < 90 mmHg (aOR, 1.14; 95% CI, 1.09–1.20; *p <* 0.001); those who had fasting blood glucose < 100 mg/dL (aOR, 1.16; 95% CI, 1.10–1.23; *p <* 0.001); those had a total cholesterol of <240 mg/dL (aOR, 1.11; 95% CI, 1.06–1.16; *p <* 0.001); males who had hemoglobin ≥ 12 g/dL and females who had hemoglobin ≥ 10 g/dL (aOR, 1.11; 95% CI, 1.06–1.16; *p <* 0.001); those who had a CCI score of 0 (aOR, 1.18; 95% CI, 1.11–1.26; *p <* 0.001) or ≥2 (aOR, 1.20; 95% CI, 1.11–1.29; *p <* 0.001); or those who did not have a history of COPD (aOR, 1.12; 95% CI, 1.07–1.18; *p <* 0.001).

## 4. Discussion

This large-scale study conducted across the country revealed a small increase in the likelihood of PD in Korean adults with asthma in comparison to those without asthma. Multivariable logistic regression analysis with adjustments for confounding factors, including demographics, socioeconomics, lifestyle, and comorbidities, revealed that asthma itself may be an independent risk factor for the development of PD, with those with asthma having an 11% higher likelihood (95% CI, 1.06–1.16). This effect of asthma on PD prevalence was consistent regardless of sex, age, residence location, alcohol consumption, or the presence of a comorbidity (obesity, hypertension, hyperglycemia, hyperlipidemia, anemia, or COPD). Our findings may help cautiously warn asthma patients of their slightly increased risk of developing PD.

The findings of two studies proposing the connection between asthma and PD risk based on the Taiwan National Health Insurance Research Database with 10,455 asthma patients and 41,820 controls (hazard ratio, 3.10; 95% CI, 2.20–4.36) [15] and a further 10,260 asthma patients and 20,513 controls (hazard ratio, 1.56; 95% CI, 1.34–1.81) [16], respectively, support our findings. Cheng et al. discovered a cumulative relationship between more severe asthma and a greater probability of PD during a 13-year longitudinal follow-up study [15]. A population-based study from the USA using Medicare beneficiaries showed that using β2-agonists as asthma therapy was linked to a lower incidence of PD [40]. This was partly backed up by subsequent tissue culture and animal experiments that showed that β2-agonists and antagonists could reduce and increase neuronal α-synuclein gene expression, respectively [41]. However, the contribution of β2-agonists or antagonists to the emergence of PD may be insignificant because β2-agonists were not associated with PD incidence in COPD patients [42]. The cohorts were heterogeneous in terms of demographics, with the majority (85%) being COPD or bronchiectasis patients and only 47% being asthma patients [40], which may limit the generalization of the connection between asthma and PD and have certain limitations due to the lack of universal healthcare databases covering the majority of the population. To reduce potential confounding effects in our study, which are more likely at older ages when PD is more common, a methodologically preferred research design with nationwide organized data was used and adjusted for potential confounders. The conclusion of an increased risk of PD following asthma was reproduced using the large number of 9029 PD individuals, who were evenly matched with 36,116 non-PD participants in the respective groups. 

The possible connections between asthma and the development of PD remain hard to pin down and may involve genetic, environmental, lifestyle, and other unidentified factors [43]. It is hypothesized that common pathophysiologies, such as hypoxemia, inflammation, oxidative stress, and immune dysfunction, may be the link between those two diseases [43]. Chronic neuroinflammation is one of the hallmarks of PD pathophysiology, since inflammation is detectable early in PD and persists throughout the disease state [43]. In rat models, the regular use of bronchodilators (e.g., β2-agonists) reduces inflammation in the airways and protects against PD [44]. Additionally, inhaled corticosteroids, which reduce airway inflammation, have been shown to lower the risk of PD development [16]. A recent study suggests that inflammation caused by particulate matter may trigger microglial activation and result in the significant death of neurons in vitro [45]. This finding implies that respiratory inflammation may contribute to the neurodegeneration associated with PD [45]. In addition, the generation of reactive free radicals that can damage macromolecules in the body, disrupting signal transduction pathways and antioxidant balance, is believed to cause diseases such as PD and asthma [46]. A gene set enrichment analysis recently revealed that PD and oxidative phosphorylation were positively associated with eosinophilic asthma [47], indicating the pathologically common gene signaling pathway between asthma and PD. Furthermore, COPD-linked single-nucleotide polymorphisms appear to control genes involved in the regulation of lung or pulmonary function, asthma, PD, brain region volumes, cortical surface area, and white matter microstructure, suggesting interactive regulatory connections, genes, and biochemical pathways that could be the cause of these occurring traits [48]. These genetic connections may explain why asthma increases the odds of developing PD in people who are not affected by other health conditions, such as obesity, hypertension, hyperglycemia, hyperlipidemia, anemia, or COPD [16]. This could mean that asthma alone may be an independent risk factor for incident PD. Therefore, PD may be one of several clinical presentations associated with asthma that share the same underlying mechanism. In addition, it is commonly seen that asthma and PD have similar comorbidities, such as dyslipidemia [49,50,51], coronary heart disease [15,52,53], and depression [15,22,54], which may imply that the two conditions have a shared pathogenesis and similar preventive treatments.

This study from being based on a representative nationwide cohort database that has balanced patients and control members due to overlap-weighted propensity score matching, which minimizes selection bias and is similar to randomized trials. Additionally, the KNHIS-HSC data includes every hospital and clinic throughout the entire country, allowing a complete medical history to be obtained during the follow-up duration, thus enhancing the generalizability and accuracy of the research findings. Furthermore, we carefully considered potential confounding variables. The full adjustment of socioeconomic status (e.g., income and area of residence), likely lifestyle-related risk factors (e.g., alcohol, blood pressure, obesity, fasting blood glucose, total cholesterol level, or hemoglobin, and smoking), and comorbidities may be a further advantage.

This study had some limitations. First, due to the observational and retrospective nature of the study design, the outcomes of this research cannot be utilized to draw a definitive connection between asthma and PD. Additionally, we did not explore the mechanisms that might explain the relationship between these two conditions. Second, given that the study only involved Korean citizens, especially adults over aged 40, and the exposure factors examined in the research were primarily obtained from Health Insurance data in the Republic of Korea, it is feasible that some unmeasured confounding variables may have remained unaccounted for, and the results may not be applicable to other populations. Third, the KNHIS-HSC database did not contain any information regarding the severity of asthma and PD, family history, personal genetics, or diets. Consequently, any missing data was not included.

## 5. Conclusions

Our findings may indicate that Korean adults who have asthma may have a slightly enhanced risk of developing PD, regardless of their demographic or lifestyle characteristics. This population-based nationwide study cautiously suggests a possible connection between asthma and incident PD in Korean adults. Additional research is necessary to validate this link and to explore the underlying mechanisms in greater depth.

## Figures and Tables

**Table 1:** General characteristics of participants before and after overlap propensity score weight.

Characteristics	Before Overlap Weighting Adjustment			After Overlap Weighting Adjustment		
	Parkinson’s Disease	Control	Standardized Difference	Parkinson’s Disease	Control	Standardized Difference
Age (n, %)			0.00			0.00
40–44	8 (0.09)	32 (0.09)		6 (0.09)	6 (0.09)	
45–49	75 (0.83)	300 (0.83)		59 (0.83)	59 (0.83)	
50–54	243 (2.69)	972 (2.69)		191 (2.68)	191 (2.68)	
55–59	514 (5.69)	2056 (5.69)		404 (5.68)	404 (5.68)	
60–64	926 (10.26)	3704 (10.26)		726 (10.20)	726 (10.20)	
65–69	1388 (15.37)	5552 (15.37)		1093 (15.36)	1093 (15.36)	
70–74	2012 (22.28)	8048 (22.28)		1583 (22.25)	1583 (22.25)	
75–79	2158 (23.90)	8632 (23.90)		1702 (23.93)	1702 (23.93)	
80–84	1299 (14.39)	5196 (14.39)		1027 (14.44)	1027 (14.44)	
85+	406 (4.50)	1624 (4.50)		322 (4.53)	322 (4.53)	
Sex (n, %)			0.00			0.00
Male	4313 (47.77)	17,252 (47.77)		3397 (47.77)	3397 (47.77)	
Female	4716 (52.23)	18,864 (52.23)		3715 (52.23)	3715 (52.23)	
Income (n, %)			0.00			0.00
1 (lowest)	1663 (18.42)	6652 (18.42)		1308 (18.38)	1308 (18.38)	
2	985 (10.91)	3940 (10.91)		777 (10.92)	777 (10.92)	
3	1206 (13.36)	4824 (13.36)		950 (13.36)	950 (13.36)	
4	1739 (19.26)	6956 (19.26)		1370 (19.26)	1370 (19.26)	
5 (highest)	3436 (38.06)	13,744 (38.06)		2708 (38.07)	2708 (38.07)	
Region of residence (n, %)			0.00			0.00
Urban	3395 (37.60)	13,580 (37.60)		2672 (37.57)	2672 (37.57)	
Rural	5634 (62.40)	22,536 (62.40)		4440 (62.43)	4440 (62.43)	
Obesity † (n, %)			0.02			0.00
Underweight	333 (3.69)	1321 (3.66)		262 (3.68)	262 (3.68)	
Normal	3183 (35.25)	12,994 (35.98)		2519 (35.41)	2519 (35.41)	
Overweight	2371 (26.26)	9445 (26.15)		1869 (26.28)	1869 (26.28)	
Obese I	2834 (31.39)	11,205 (31.03)		2224 (31.28)	2224 (31.28)	
Obese II	308 (3.41)	1151 (3.19)		238 (3.35)	238 (3.35)	
Smoking status (n, %)			0.09			0.00
Non-smoker	7005 (77.58)	26,777 (74.14)		5471 (76.92)	5471 (76.92)	
Past smoker	1202 (13.31)	5186 (14.36)		964 (13.55)	964 (13.55)	
Current smoker	822 (9.10)	4153 (11.50)		678 (9.53)	678 (9.53)	
Alcohol consumption (n, %)			0.10			0.00
<1 time a week	6544 (72.48)	24,535 (67.93)		5090 (71.57)	5090 (71.57)	
≥1 time a week	2485 (27.52)	11,581 (32.07)		2022 (28.43)	2022 (28.43)	
SBP (Mean, SD)	129.86 (17.41)	129.96 (16.90)	0.01	129.88 (15.44)	129.88 (7.52)	0.00
DBP (Mean, SD)	78.33 (10.78)	78.26 (10.51)	0.01	78.31 (9.55)	78.31 (4.69)	0.00
FBG (Mean, SD)	106.82 (36.23)	103.59 (29.56)	0.10	105.86 (29.91)	105.86 (15.27)	0.00
Total cholesterol (Mean, SD)	193.89 (43.68)	195.53 (40.55)	0.04	194.24 (39.21)	194.24 (17.77)	0.00
Hemoglobin (Mean, SD)	13.45 (1.49)	13.52 (1.49)	0.05	13.47 (1.32)	13.47 (0.67)	0.00
CCI score (Mean, SD)	1.73 (1.90)	1.24 (1.81)	0.27	1.62 (1.61)	1.62 (0.95)	0.00
COPD (n, %)	1198 (13.27)	4235 (11.73)	0.05	920 (12.94)	920 (12.94)	0.00
Asthma (n, %)	2624 (29.06)	9697 (26.85)	0.05	2062 (28.99)	1930 (27.13)	0.04

Abbreviations: CCI, Charlson Comorbidity Index; SBP, Systolic blood pressure; DBP, Diastolic blood pressure; FBG, Fasting blood glucose; COPD, chronic obstructive pulmonary disease. † Obesity (BMI, body mass index, kg/m^2^) was categorized as <18.5 (underweight), ≥18.5 to <23 (normal), ≥23 to <25 (overweight), ≥25 to <30 (obese I), and ≥30 (obese II).

**Table 2:** Crude and overlap propensity score-weighted odds ratios of asthma for Parkinson’s disease.

Characteristics	N of Parkinson’s Disease	N of Control	Odds Ratios for Parkinson’s Disease (95% Confidence Interval)			
	(Exposure/Total, %)	(Exposure/Total, %)	Crude †	*p*-Value	Overlap-Weighted Model †	*p*-Value
Total participants (n = 45,145)						
Non-asthma	6405/9029 (70.9)	26,419/36,116 (73.2)	1		1	
Asthma	2624/9029 (29.1)	9697/36,116 (26.8)	1.12 (1.06–1.17)	<0.001 *	1.11 (1.06–1.16)	<0.001 *
Age < 75 years old (n = 25,830)						
Non-asthma	3876/5166 (75.0)	16,034/20,664 (77.6)	1		1	
Asthma	1290/5166 (25.0)	4630/20,664 (22.4)	1.15 (1.07–1.24)	<0.001 *	1.15 (1.08–1.22)	<0.001 *
Age ≥ 75 years old (n = 19,315)						
Non-asthma	2529/3863 (65.5)	10,385/15,452 (67.2)	1		1	
Asthma	1334/3863 (34.5)	5067/15,452 (32.8)	1.08 (1.00–1.16)	0.04 *	1.07 (1.00–1.14)	0.041 *
Male (n = 21,565)						
Non-asthma	3183/4313 (73.8)	13,110/17,252 (76.0)	1		1	
Asthma	1130/4313 (26.2)	4142/17,252 (24.0)	1.12 (1.04–1.21)	0.003 *	1.12 (1.05–1.19)	0.001 *
Female (n = 23,580)						
Non-asthma	3222/4716 (68.3)	13,309/18,864 (70.6)	1		1	
Asthma	1494/4716 (31.7)	5555/18,864 (29.4)	1.11 (1.04–1.19)	0.003 *	1.09 (1.03–1.16)	0.002 *
Low income group (n = 19,270)						
Non-asthma	2758/3854 (71.6)	11,282/15,416 (73.2)	1		1	
Asthma	1096/3854 (28.4)	4134/15,416 (26.8)	1.08 (1.00–1.17)	0.043 *	1.06 (0.99–1.13)	0.101
High income group (n = 25,875)						
Non-asthma	3647/5175 (70.5)	15,137/20,700 (73.1)	1		1	
Asthma	1528/5175 (29.5)	5563/20,700 (26.9)	1.14 (1.07–1.22)	<0.001 *	1.15 (1.08–1.21)	<0.001 *
Urban resident (n = 16,975)						
Non-asthma	2430/3395 (71.6)	10,017/13,580 (73.8)	1		1	
Asthma	965/3395 (28.4)	3563/13,580 (26.2)	1.12 (1.03–1.21)	0.01 *	1.12 (1.04–1.20)	0.003 *
Rural resident (n = 28,170)						
Non-asthma	3975/5634 (70.6)	16,402/22,536 (72.8)	1		1	
Asthma	1659/5634 (29.4)	6134/22,536 (27.2)	1.12 (1.05–1.19)	<0.001 *	1.10 (1.05–1.17)	<0.001 *

* Significance at *p* < 0.05. † Adjusted for age, sex, income, region of residence, obesity, smoking, alcohol consumption, SBP, DBP, fasting blood glucose, total cholesterol, hemoglobin, CCI scores, and COPD.

**Table 3:** Subgroup analyses of crude and overlap propensity score-weighted odds ratios of asthma for Parkinson’s disease.

Characteristics	N of Parkinson’s Disease	N of Control	Odds Ratios for Parkinson’s Disease (95% Confidence Interval)			
	(Exposure/Total, %)	(Exposure/Total, %)	Crude †	*p*-Value	Overlap-Weighted Model †	*p*-Value
Obesity						
Underweight (n = 1654)	88/333 (26.4)	379/1321 (28.7)	0.89 (0.68–1.17)	0.412	0.94 (0.74–1.20)	0.623
Normal (n = 16,177)	904/3183 (28.4)	3246/12,994 (25.0)	1.19 (1.09–1.30)	<0.001 *	1.20 (1.12–1.29)	<0.001 *
Overweight (n = 11,816)	637/2371 (26.9)	2471/9445 (26.2)	1.04 (0.94–1.15)	0.484	1.00 (0.92–1.09)	0.995
Obese (n = 15,498)	995/3142 (31.7)	3601/12,356 (29.1)	1.13 (1.04–1.23)	0.006 *	1.12 (1.04–1.20)	0.003 *
Smoking status						
Non-smoker (n = 33,782)	2062/7005 (29.4)	7370/26,777 (27.5)	1.10 (1.04–1.16)	0.001 *	1.11 (1.05–1.16)	<0.001 *
Past smoker (n = 6388)	330/1202 (27.5)	1398/5186 (27.0)	1.03 (0.89–1.18)	0.726	1.02 (0.90–1.15)	0.793
Current smoker (n = 4975)	232/822 (28.2)	929/4153 (22.4)	1.36 (1.15–1.62)	<0.001 *	1.25 (1.09–1.44)	0.002 *
Alcohol consumption						
<1 time a week (n = 31,079)	1924/6544 (29.4)	6725/24,535 (27.4)	1.10 (1.04–1.17)	0.001 *	1.11 (1.05–1.17)	<0.001 *
≥1 time a week (n = 14,066)	700/2485 (28.2)	2972/11,581 (25.7)	1.14 (1.03–1.25)	0.010 *	1.10 (1.02–1.19)	0.020 *
Blood pressure (mmHg)						
SBP < 140 and DBP < 90 (n = 31,370)	1874/6223 (30.1)	6826/25,147 (27.1)	1.16 (1.09–1.23)	<0.001 *	1.14 (1.09–1.20)	<0.001 *
SBP ≥ 140 or DBP ≥ 90 (n = 13,775)	750/2806 (26.7)	2871/10,969 (26.2)	1.03 (0.94–1.13)	0.550	1.03 (0.95–1.12)	0.453
Fasting blood glucose						
<100 mg/dL (n = 25,211)	1422/4758 (29.9)	5451/20,453 (26.7)	1.17 (1.09–1.26)	<0.001 *	1.16 (1.10–1.23)	<0.001 *
≥100 mg/dL (n = 19,934)	1202/4271 (28.1)	4246/15,663 (27.1)	1.05 (0.98–1.14)	0.179	1.05 (0.98–1.12)	0.175
Total cholesterol						
<240 mg/dL (n = 39,364)	2290/7862 (29.1)	8498/31,502 (27.0)	1.11 (1.05–1.18)	<0.001 *	1.11 (1.06–1.16)	<0.001 *
240 mg/dL (n = 5781)	334/1167 (28.6)	1199/4614 (26.0)	1.14 (0.99–1.32)	0.069	1.11 (0.98–1.25)	0.100
Hemoglobin ≥ (g/dL)						
≥12 for men and ≥10 for women (n = 43,653)	2521/8695 (29.0)	9363/34,958 (26.8)	1.12 (1.06–1.18)	<0.001 *	1.11 (1.06–1.16)	<0.001 *
<12 for men and <10 for women (n = 1492)	103/334 (30.8)	334/1158 (28.8)	1.10 (0.84–1.43)	0.480	1.06 (0.83–1.36)	0.629
CCI scores						
0 (n = 21,447)	820/2950 (27.8)	4592/18,497 (24.8)	1.17 (1.07–1.27)	<0.001 *	1.18 (1.11–1.26)	<0.001 *
1 (n = 8638)	568/2177 (26.1)	1937/6461 (30.0)	0.82 (0.74–0.92)	<0.001 *	0.86 (0.77–0.95)	0.002 *
≥2 (n = 15,060)	1236/3902 (31.7)	3168/11,158 (28.4)	1.17 (1.08–1.27)	<0.001 *	1.20 (1.11–1.29)	<0.001 *
COPD history						
No (n = 39,712)	1880/7831 (24.0)	7088/31,881 (22.2)	1.11 (1.04–1.17)	<0.001 *	1.12 (1.07–1.18)	<0.001 *
Yes (n = 5433)	744/1198 (62.1)	2609/4235 (61.6)	1.02 (0.89–1.17)	0.755	1.04 (0.93–1.16)	0.475

Abbreviations: CCI, Charlson Comorbidity Index; SBP, Systolic blood pressure; DBP, Diastolic blood pressure; COPD, chronic obstructive pulmonary disease. * Significance at *p* < 0.05. † Adjusted for age, sex, income, region of residence, obesity, smoking, alcohol consumption, SBP, DBP, fasting blood glucose, total cholesterol, hemoglobin, CCI scores, and COPD.

## Data Availability

All data are available from the database of National Health Insurance Sharing Service (NHISS) https://nhiss.nhis.or.kr/ (accessed on 1 May 2022). NHISS allows access to all of this data for the any researcher who promises to follow the research ethics at some processing charge. If you want to access the data of this article, you can download it from the website after promising to follow the research ethics.
